# Loss of Receptor on Tuberculin-Reactive T-Cells Marks Active Pulmonary Tuberculosis

**DOI:** 10.1371/journal.pone.0000735

**Published:** 2007-08-15

**Authors:** Mathias Streitz, Lydia Tesfa, Vedat Yildirim, Ali Yahyazadeh, Timo Ulrichs, Rodica Lenkei, Ali Quassem, Gerd Liebetrau, Laurel Nomura, Holden Maecker, Hans-Dieter Volk, Florian Kern

**Affiliations:** 1 Institut für Medizinische Immunologie der Charité, Charité Universitätsmedizin Berlin, Berlin, Germany; 2 Abteilung Immunologie, Max-Planck-Institut für Infektionsbiologie, Berlin, Germany; 3 Capio Diagnostik AB, St Görans Hospital, Stockholm, Sweden; 4 Lungenklinik Lostau, Lostau, Germany; 5 BD Biosciences, San Jose, California, United States of America; 6 Division of Medicine, Brighton and Sussex Medical School, Brighton, United Kingdom; University of California at San Francisco, United States of America

## Abstract

**Background:**

Tuberculin-specific T-cell responses have low diagnostic specificity in BCG vaccinated populations. While subunit-antigen (e.g. ESAT-6, CFP-10) based tests are useful for diagnosing latent tuberculosis infection, there is no reliable immunological test for active pulmonary tuberculosis. Notably, all existing immunological tuberculosis-tests are based on T-cell response size, whereas the diagnostic potential of T-cell *response quality* has never been explored. This includes surface marker expression and functionality of mycobacterial antigen specific T-cells.

**Methodology/Principal Findings:**

Flow-cytometry was used to examine over-night antigen-stimulated T-cells from tuberculosis patients and controls. Tuberculin and/or the relatively *M. tuberculosis* specific ESAT-6 protein were used as stimulants. A set of classic surface markers of T-cell naïve/memory differentiation was selected and IFN-γ production was used to identify T-cells recognizing these antigens. The percentage of tuberculin-specific T-helper-cells lacking the surface receptor CD27, a state associated with advanced differentiation, varied considerably between individuals (from less than 5% to more than 95%). Healthy BCG vaccinated individuals had significantly fewer CD27-negative tuberculin-reactive CD4 T-cells than patients with smear and/or culture positive pulmonary tuberculosis, discriminating these groups with high sensitivity and specificity, whereas individuals with latent tuberculosis infection exhibited levels in between.

**Conclusions/Significance:**

Smear and/or culture positive pulmonary tuberculosis can be diagnosed by a rapid and reliable immunological test based on the distribution of CD27 expression on peripheral blood tuberculin specific T-cells. This test works very well even in a BCG vaccinated population. It is simple and will be of great utility in situations where sputum specimens are difficult to obtain or sputum-smear is negative. It will also help avoid unnecessary hospitalization and patient isolation.

## Introduction

The goal of this work was to establish a new biomarker for clinical tuberculosis based on measurable changes that TB specific CD4 T-cells undergo as they differentiate in response to virulent *mycobacteria*. The diagnosis of acute tuberculosis is usually based on characteristic X-ray findings, clinical symptoms, and positive sputum-smear or culture. However, even if the sputum-smear is negative, patients suspected to have active pulmonary TB will frequently be hospitalized and treated until culture results are obtained. This is an unnecessary burden on the patient and public spending if cultures turn out negative. Unfortunately, T-cell responses to tuberculin have no diagnostic value in BCG vaccinees unless very strongly positive. Meanwhile, T-cell responses to the relatively *M. tuberculosis* specific ESAT-6 protein are the gold standard in the detection of latent tuberculosis infection (more recently in connection with other culture filtrate proteins) [1]–[5], but were reported not to be reliable for the diagnosis of acute TB [6], [7]. As a matter of fact, a rapid and reliable immunological test for active pulmonary tuberculosis does not exist to date.

The role of T-cells in controlling TB is dramatically illustrated by the HIV pandemic. Whereas only 10% of latently TB infected but otherwise healthy individuals progress to active TB in their lifetime, this figure increases to 10% per year in HIV infected individuals [8]. This observation is strong evidence that CD4 T-helper-cells, which are gradually lost in HIV infection, have a central role in controlling TB. Upon activation they can release IFN-γ, which drives monocytes to destroy intracellular *mycobacteria*. The subsequent production of TNF-α by monocytes is essential for containing *mycobacteria* inside granulomas [9]. Various other immune cells including CD8 T-cells appear to contribute to the control of TB [10].

Since CD4 T-cells are so important in the defence against TB, we were hoping to find differences in the degree of differentiation resulting from the ‘one-off’ stimulus provided by BCG vaccination on the one hand and the exposure to virulent replicating *mycobacteria* on the other. Given such differences, this new type of test would also be able to be used in a BCG-vaccinated population, where tuberculin based tests are normally difficult to use. In this and our previous studies [11], the T-cells that responded by production of IFN-γ, TNF-α, or IL-2 to short term (16h) ex-vivo stimulation with tuberculin (purified protein derivate, or PPD) or the early antigenic target protein 6 (ESAT-6), largely overlapped in regards of cytokine production (with respect to each antigen) and were almost exclusively CD4-positive. This was an additional reason why we focused on this subset and IFN-γ production.

Patterns of antigen-dependent T-cell differentiation have been a central issue in T-cell immunology for a number of years both in humans and animal models [12]–[15]. Changes in surface marker expression reflect changes in T-cell function, circulation pathways, and co-stimulation requirements, for example. Such changes can be visualized very efficiently by flow-cytometry [16] and, as a result, this method has been very commonly used to research HIV-specific T-cells. However, it was not until recently that it was first applied to TB [6], [11], [17], [18].

CD27 is a receptor for co-stimulation expressed on the T-cell surface and down-regulated when T-cells progress from a non-antigen experienced towards a terminal memory stage. It was recently shown that IFN-γ producing CD4 T-cells in the lungs of TB infected mice are predominantly CD27-negative [19]. IFN-γ being an important player in the immune response against TB [9], these CD27-negative CD4 T-cells may be instrumental in controlling infection. To build on this discovery, the distribution of CD27 on IFN-γ producing tuberculin-reactive CD4 T-cells in TB infected humans was the central parameter in our study. This work was done on blood cells rather than pulmonary lymphocytes; however, we discovered that the measurement of CD27 expression on tuberculin-reactive human CD4 T-cells is a useful and rapid tool for the diagnosis of active pulmonary tuberculosis. Results can be obtained in less than 24 hours.

## Methods

### Patients and patient materials

*Smear and/or culture positive pulmonary TB* was established by positive microscopy and/or culture of sputum and/or bronchio-alveolar lavage (BAL) fluid. *Smear and culture negative Pulmonary TB* refers to TB diagnosed in the absence of positive sputum microscopy or culture, based on X-ray lesions, positive histology of biopsy materials, and/or response to TB therapy. All individuals recruited (TB patients, control patients, healthy donors) had been BCG-vaccinated in early childhood unless specifically stated as for some of the latently infected individuals (BCG vaccination being obligatory in the former German Democratic Republic of which almost all recruited patients used to be citizens). None of the tested patients or controls were known or suspected to be HIV-seropositive. HIV serostatus was routinely obtained in TB patients but not the other donors. All patients were recruited at Lungenklinik Lostau, Lostau, Germany between 1/2005 and 10/2006. Samples were transported within 4 hours of collection to the Institute for Medical Immunology, Charité – Universitaetsmedizin Berlin, Berlin, Germany.

Blood from more than 30 patients with TB at different stages of treatment was used to establish antibody staining panels using tuberculin and/or ESAT-6 for stimulation. An initial comparative and retrospective study focused on the distribution of CD27 on tuberculin-reactive CD4 T-cells and included 6 patients with open pulmonary TB (4 female, 2 male, age 23–79 years) and 31 healthy individuals with no signs or symptoms of TB but various degrees of exposure (household contacts, health care workers in a lung hospital, medical students, and unexposed healthy blood donors; 23 female, 8 male, age 22–69 years). Within this group 5 individuals had latent TB infection, 17 were highly exposed, and 9 were considered unexposed. The diagnosis of latent TB infection was established by a positive response to tuberculin in non-BCG vaccinated individuals (n = 3). Two individuals who had shared their bed with a patient with smear positive TB where also classified as having latent TB. In individuals classified as ‘highly exposed’ latent TB infection had not been conclusively demonstrated but for some of them would be suggested by their very close and continued exposure. These included hospital personnel with regular direct contact with TB, including some regularly performing BAL on TB patients.

**Figure 1:**
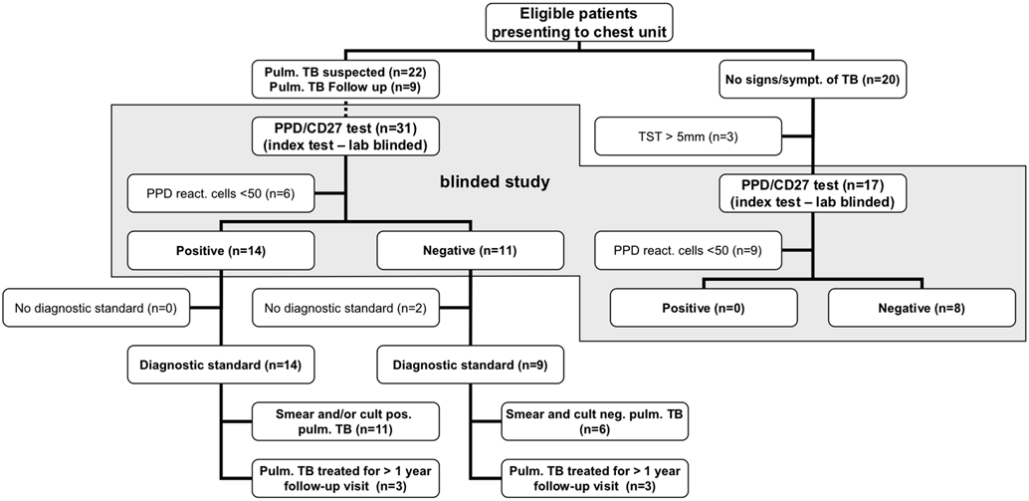
Flow diagram of the blind prospective diagnostic study. Eligible patients were recruited by the admitting physician. Diagnostic standard refers to the standard diagnostic procedure for TB in this hospital including history, X-ray, sputum microscopy and culture, PCR, biopsy and histology where applicable. TST = tuberculin skin test

A blinded prospective study was designed to confirm the results of the initial study. The diagnoses relating to coded samples were not revealed to the lab until results had been reported back in print to the clinicians obtaining and coding the samples. For this study 31 additional patients with smear and/or culture positive or smear and culture negative pulmonary TB (11 female, 20 male, median age 51 years, range 15–96 years) were recruited within two months of treatment initiation, 9 patients at follow-up at least one year after beginning of therapy, and 20 control patients with no known history of or exposure to TB (6 female, 14 male, age 40–76 years). Disease severity in TB patients was based on the number and size of lesions observed on X-ray, however, these data were not used to subgroup patients. All TB patients except for one had more than one lesion on X-ray with at least one lesion larger than 3 cm diameter. Treatment was based on triple (isoniazid, ethambutol, and rifampicin) or quadruple (triple plus pyrazinamide) regimen. It was continued for one year in all patients and prolonged depending on follow-up evaluation. In most cases, TB culture results were pending at the time of sample collection for flow-cytometry whereas a sputum smear had been obtained. Blood was drawn into citrated blood collection tubes following written informed consent as approved by the Charité Ethics Committee. The study protocol was in accordance with the Declaration of Helsinki. A flow diagram of the study is presented in Fig. 1.

### Cell preparation and cytokine induction assay

This method was described in detail elsewhere [11]. Samples were processed immediately, within 4 hours of blood collection. Briefly, aliquots of 400 µl of PBMC suspended in complete culture media (5×10^6^ cells/ml) were placed in 5 ml polypropylene tubes (BD, Heidelberg, Germany). Stimulants were added in a volume of 100 µl (1 µg/ml final concentration for each of 21 ESAT-6 peptides or 10 µl/ml final concentration of tuberculin (PPD, SSI, Copenhagen, Denmark)). After 2 hours of incubation (standard incubator) 2 µl Brefeldin A (5 mg/ml working solution in DMSO) was added and the volume adjusted to 1 ml (culture media). After 16 hours ice-cold PBS (3 ml) was added and tubes were centrifuged. Supernatants were removed, pellets were resuspended (2mM EDTA in PBS) and incubated (37°C water bath, 10 min). Adherent cells were detached by two subsequent wash/centrifugation steps (0.5 % (w/v) BSA (Biochrom, Germany) and 0.1% sodium-azide (Serva, Heidelberg, Germany) in PBS) and intermittent vortexing (30s). Pellets were resuspended for antibody surface staining (30 min, dark, on ice), followed by fixation, permeabilization (BD Lysing Solution and Permeabilizing solution 2; used according to the manufacturer's instructions), and intracellular staining (see surface staining).

### Monoclonal antibodies

Pacific-blue (PB)-labeled anti-CD3, AmCyan-labeled anti-CD4, Fluorescein-Iso-Thio-Cyanate (FITC)-labeled anti-IL2, anti-CD27, and anti-CD28, Phycoerythrin(PE)-labeled anti-IL-2 and anti-CD62L, Peridine-Chlorophyll(PerCP)-labeled anti-CD3, Allophyocyanine(APC)-labeled anti-IFN-γ were purchased from BD. PE-Texas-Red-(ECD)-labeled anti-CD4 and Phycoerythrin(PE)-labeled anti-CD57 where purchased from Beckman-Coulter (Krefeld, Germany), PE-Cy7-labeled anti-CD8 was purchased from Caltag (Hamburg, Germany). Fluorochrome-labeled isotype controls were used as appropriate.

### Flow-cytometric analysis

Flow-cytometry was performed on an LSR II flow-cytometer (BD). At least 250,000 lymphocytes were acquired per tube. Data files were analyzed using FACSDiva™ software (BD). The percentage of IFN-γ-positive CD4 T-cells was determined by a standardized strategy. The median number of positive events evaluated was 479. Given 479 events the 95%-confidence interval for a proportion of CD27-negative events determined to be 50% would be 50+/− 4.5% (given normal distribution). Distributions based on a minimum of 50 and 20 events were considered for tuberculin and ESAT-6 stimulated samples, respectively, the latter only when comparing the distribution of tuberculin and ESAT-6 specific CD4 T-cells. Whether more than 50 tuberculin-reactive CD4 T-cells could be collected depended on the patient lymphocyte count, reactivity, and the number of stimuli and antibody panels tested.

### Peptides and antigens

The ESAT-6 peptide set was made in-house (21 peptides, 15 amino-acid length, 11 overlaps between peptides, covering the Swiss-Prot Q57165 sequence). Peptides were dissolved in DMSO and used as described above. Tuberculin (PPD) was purchased from SSI (Copenhagen, Denmark).

### Cytokine measurements

Supernatants of overnight stimulated samples (same conditions as above save addition of Brefeldin A) were stored at −80°C until measurement using a custom ordered bead-array (Lincoplex™) by Linco Research (Stockholm, Sweden) and a Luminex-xMAP System.

### Personnel involved in grouping patients, preparing, reading, and analysing samples

The reference laboratory tests (smear and culture) were performed by fully trained hospital and laboratory personnel (routine TB diagnostic lab). Biopsies were analysed by a fully trained experienced pathologist. X-rays were evaluated by fully trained and experienced chest physicians/radiologists. All samples were prepared and measured by 2 laboratory scientists with long-term experience in flow-cytometry. Flow-cytometric data analysis was performed by a highly experienced medical scientist.

### Statistical analysis

SPSS 11.0 (SPSS Inc, Chicago, USA) software was used. Differences between paired samples were analysed using the Wilcoxon test. Receiver Operating Characteristic (ROC)-analysis was used to define cut-off values. Differences between unpaired sample were tested using the Mann-Whitney U-test.

## Results

### Initial observation: marked down-regulation of CD27 on tuberculin-specific T-cells in active pulmonary TB

**Figure 2:**
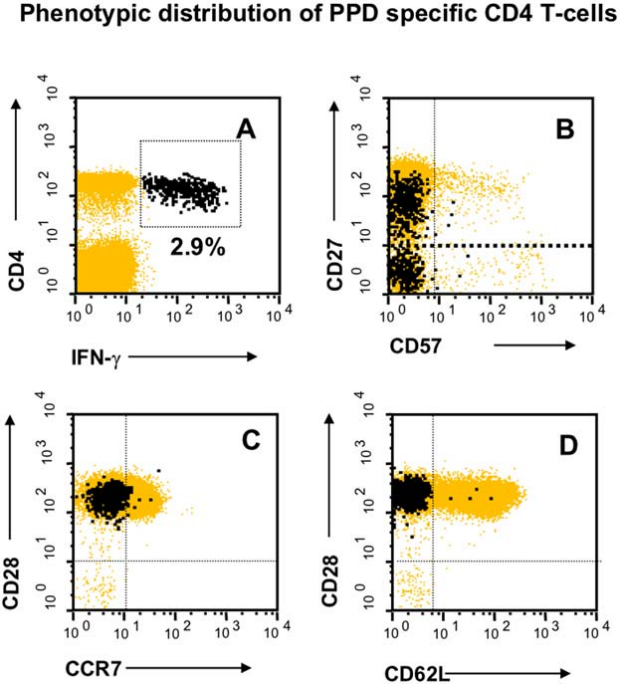
Distribution of tuberculin-reactive CD4 T-cells among the subsets defined by classic memory/naïve markers. PBMC were stained with the indicated monoclonal antibodies. Panel A shows CD3 T-cells, panels B-D only CD4 T-cells. IFN-γ-producing ex-vivo tuberculin-reactive events are highlighted black. The dotted horizontal line in B shows the limit set for CD27-positivity. Representative results from one patient with active TB are shown.

**Figure 3:**
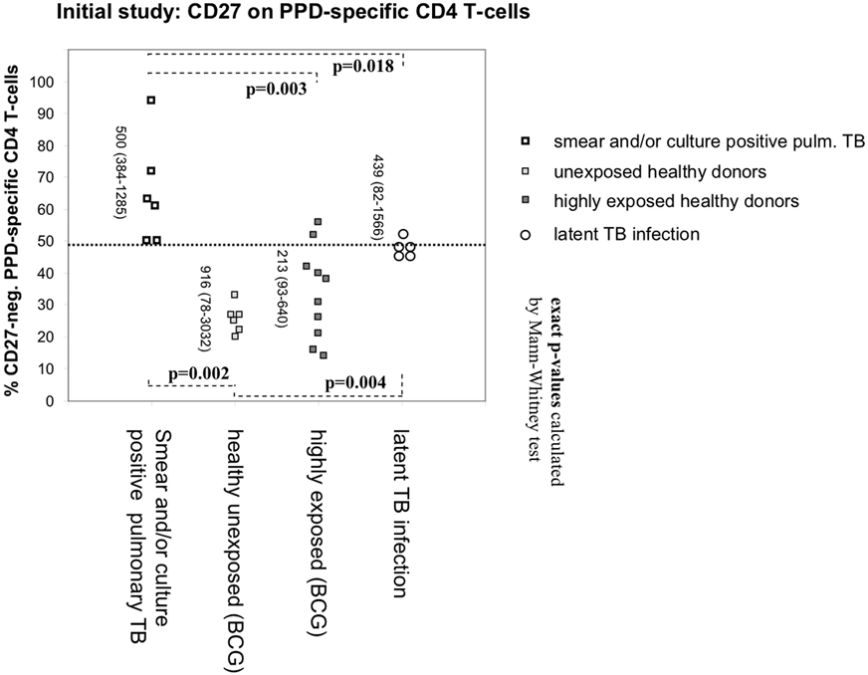
Significantly fewer tuberculin-reactive CD4 T-cells express CD27 in TB-patients than in controls. Proportions of CD27-positive and negative cells were based on a minimum of 50 IFN-γ positive events. Vertical numbers indicate evaluated events (median and range). Controls included unexposed controls, professionally TB-exposed health care workers, and donors with latent infection. A threshold of 49% would effectively discriminate between patients and controls (dotted line). Controls with latent TB infection had higher values than individuals with no known exposure to TB.

The expression of “classic” markers of T-cell differentiation [12], [14] on tuberculin-reactive CD4 T-cells in a representative patient with smear and/or culture positive TB is shown in Figure 2. Expression of CD27 was high, intermediate, or absent (black highlighted events in panel B), expression of the chemokine receptor, CCR7, and the adhesion molecule, L-Selectin (CD62L), was generally absent, and expression of the costimulatory receptor, CD28, was generally high (panels C and D). Expression of both the traditional memory markers, CD45RA and RO, was intermediate (not shown). As previously reported [11], the majority of IFN-γ-producing tuberculin-reactive CD4 T-cells also produced TNF-α and IL-2 (not shown). The phenotype of tuberculin-reactive T-helper cells was similar in the control group (BCG vaccines) with one exception: the percentage of CD27-negative tuberculin-reactive CD4 T-cells was clearly higher in the patients. In particular, there was a significant difference between patients with smear and/or culture positive pulmonary TB and unexposed controls (Fig.3). Five individuals classified as having latent TB infection had values in between the patients and the unexposed/highly exposed controls (Fig. 3).

To be statistically viable, results shown in Figure 3 only include percentages based on *more than 50 IFN-γ producing cells* (median: 479 cells). Receiver-Operating-Characteristics (ROC)-analysis identified a threshold of 49% (percentage of CD27-negative/IFN-γ-positive tuberculin-reactive CD4 T-cells) that best discriminated patients and controls (sensitivity = 100%, specificity = 85.7%, positive predictive value = 100%, negative predictive value = 94%). Notably, there was no correlation between the reactivity to tuberculin (in % reactive cells or IFN-γ levels in the supernatant) and the proportion of CD27-negative T-cells (not shown). This initial observation suggested that the percentage of CD27-expressing TB specific T-cells decreases as a function of increasing TB exposure, with a percentage that is high in unexposed individuals, intermediate in latent TB infection, and low in smear and/or culture positive TB.

### Blinded evaluation confirms the utility of measuring CD27 on tuberculin reactive T-cells

To further explore the possibility of diagnosing TB based on the CD27-expression of TB specific T-helper-cells, a prospective *blinded* study was designed. To exclude an influence of the clinical diagnosis on sample analysis (e.g. the boundary dividing CD27-pos. and –neg. cells), samples received in the lab were number-coded. Clinical information was not given to the lab investigators until results had been transmitted to the clinicians in print. For this study, patients with pulmonary TB were included no later than 2 months after beginning of tuberculosis therapy (n = 22, fresh TB) or at the time of follow-up 444 +/− 94 days (mean +/− STD) after beginning of treatment (n = 9, follow-up). They were divided into, first, smear and/or culture positive pulmonary TB (n = 12, group I), second, smear and culture negative pulmonary TB (n = 10, group II), and third, pulmonary TB treated at follow up who had been treated for one year (n = 9, group III). Patients with other pulmonary or cardiovascular complaints were used as controls (n  = 20, group IV). A tuberculin skin test (TST) was performed on a majority of the control patients and those with a positive result >5 mm were excluded (n = 3). Please see Table 1 for details on all groups.

**Table 1:** Patients included in the blind study.

Dx^a^	code	Age (years)	sex	TST (mm)	Tuberculin stimulated (10 µg per ml)		ESAT-6 stimulated (20 µg of peptide per ml)	
					CD4 T-cells (%)^b^	IFN-γ (IU)^c^	CD4 T-cells (%)^b^	FN-γ(IU)^c^
**Patients with smear and/or culture positive pulmonary TB**								
1	B1	45.94	m	6	0.1430	4.90	0.0158	0.15
1	B3	81.57	f	10	0.2962	1.33	0.0000	nd
1	B11	32.96	m	nd	0.1833	3.77	0.0066	0.00
1	B12	50.63	f	10	0.1298	4.00	0.0029	0.00
1	B33	31.85	f	nd	0.0143	0.69	0.0010	0.00
1	B48	66.23	m	nd	0.0740	1.42	0.0005	0.00
1	B49	14.99	m	<5	0.1031	10.59	0.0007	0.00
1	B52	71.11	m	<5	0.1374	1.10	**0.0143**	0.21
1	B61	28.51	m	nd	0.1411	9.03	**0.0494**	1.46
1	B75	75.85	f	35	0.3035	10.23	Nd	1.00
1	B76	95.69	m	nd	1.2005	4.08	**0.0121**	0.01
1	B77	28.64	f	5	0.8713	44.79	**0.0439**	1.83
Patients with smear and culture negative pulmonary TB								
3	B21	35.33	f	20	0.1405	1.99	0.0023	0.00
2	B29	69.58	m	<5	0.0012	0.00	0.0013	0.00
2	B40	50.41	m	10	0.3424	12.89	0.0003	0.00
2	B42	41.97	m	28	0.1359	5.86	0.0005	0.00
4	B46	71.33	m	30	0.5766	13.37	**0.0360**	0.69
2	B47	66.7	m	<5	0.0200	0.04	0.0043	0.00
2	B59	64.73	m	5	0.0926	0.43	0.0012	0.00
2	B65	74.38	m	nd	0.0034	0.00	0.0049	0.00
2	B70	70.99	f	<5	0.0026	0.03	0.0016	0.00
2	B73	15.82	m	10	0.0145	0.00	0.0046	0.00
Patients with pulmonary TB at least one year after beginning of treatment								
2	B7	21.22	m	12	0.1996	6.59	**0.0209**	0.41
1	B22	50.24	f	nd	0.3246	16.66	0.0023	0.12
1	B27	58.56	m	16	0.2539	7.79	0.0018	0.00
1	B43	76.38	f	12	0.7997	3.23	0.0049	0.01
1	B51	36.46	m	nd	0.0221	nd	0.0222	nd
1	B56	61.23	f	<5	0.0417	0.24	**0.0178**	0.15
1	B57	60.11	m	<5	0.0788	2.18	0.0006	0.00
1	B58	37.47	f	25	0.0308	2.41	**0.0252**	0.26
1	B60	49.69	m	5	0.0764	2.59	0.0009	0.00
Control patients								
6	B8	44.68	f	<5	0.0151	0.00	0.0039	0.00
6	B10	58.92	m	<5	0.0471	0.00	**0.0161**	0.00
6	B15	71.3	m	<5	0.0252	0.01	0.0001	nd
6	B16	55.11	f	nd	0.0022	0.02	nd	0.00
6	B17	40.84	m	<5	0.1161	1.00	0.0030	0.00
6	B18	65.85	f	nd	0.0613	0.25	**0.0121**	nd
6	B23	70.01	m	<5	0.2552	2.55	0.0060	0.00
6	B24	42.35	m	<5	0.0273	0.02	0.0046	0.00
6	B25	76.07	f	<5	0.0255	0.00	0.0012	0.00
6	B26	69.91	f	nd	0.0086	0.00	0.0000	7.12
6	B28	64.3	m	<5	0.0078	0.23	0.0007	0.00
6	B35	48.05	m	<5	0.0058	0.27	0.0013	0.00
6	B36	58.67	m	nd	0.0088	0.16	0.0063	0.00
6	B39	47.24	m	<5	0.3653	6.06	0.0040	0.72
6	B41	43.94	m	<5	0.0019	0.18	0.0005	0.00
6	B44	73.7	m	nd	0.1972	0.50	**0.0243**	0.13
6	B55	54.19	m	<5	0.0035	0.01	0.0017	0.00

a mainstay of diagnosis: 1 = positive culture and/or microscopy, 2 = mycobacteria in biopsy, 3 = therapy response, 4 = typical chest film, TST >15 mm, positive response to ESAT-6

b results exceeding 1 positive event per 10,000 cells were counted as positive and are shown in bold

c some of these results exceed the threshold for positivity published for a commercial assay (Quantiferon Gold, Cellestis) of 0.35 IU.

**Figure 4:**
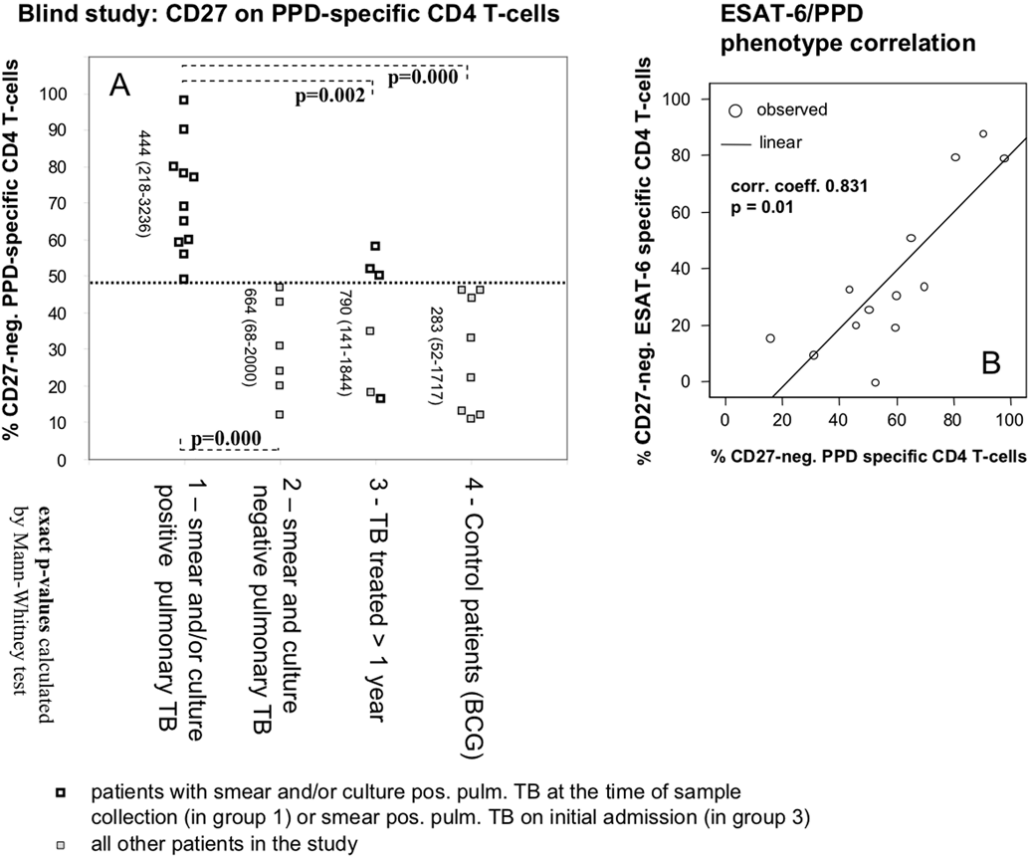
Loss of CD27 on tuberculin-reactive CD4 T-cells identifies smear and/or culture positive TB. A: Patients with smear and/or culture positive pulmonary TB were distinguishable from those with smear and culture negative pulmonary TB using a threshold of 48% (dotted line). Patients more than 1 year after beginning of treatment mostly had values >48% if TB was initially smear positive (empty squares), and <48%, if TB was smear negative (filled squares). Statistically significant differences are indicated (Chi-square test). Primary end-point was the percentage of CD27-negative cells among reactive cells. Vertical numbers indicate the number of evaluated events (median and range). B: Loss of CD27 expression on tuberculin and ESAT-6 specific CD4 T-cells is closely correlated.

The blind study confirmed the findings of the initial study (Figure 4 A). Patients with smear and/or culture positive TB could be readily discriminated from control patients, but also, from patients with smear and culture negative TB (group 2). Using a threshold of 48% (determined by ROC-analysis) above which cases would be diagnosed to have smear and/or culture positive TB, both sensitivity and specificity would be 100% (p = 0.000 in both cases, Chi-square test); also, positive and negative predictive values would be 100%. Using the original threshold of 49% suggested by the initial study, there would have been one false negative per group comparison. No distinction was possible between groups 2, 3, and 4. Table 2 shows a cross tabulation of results by patient group.

**Table 2:** Crosstabulation of results in blind study.

Group	CD27-neg. tuberculin reactive CD4 T-cells		Total
	< 48%	>48%	
1	0	11	11
2	6	0	6
3	3	3	6
4	8	0	8
Total	17	14	31

Figure 4 B shows the correlation between the percentages of CD27-negative IFN-γ producing CD4 T-cells in response to tuberculin and ESAT-6 stimulation, suggesting very similar expression levels and distribution of CD27 on the responding T-cells. There was no significant correlation between the magnitudes (frequencies) of tuberculin and ESAT-6 specific CD4 T-cell responses between any of the groups.

As in the initial study, reactivity to tuberculin measured either by IFN-γ in the supernatant or the frequency of tuberculin-reactive CD4 T-cells would not have been able to discriminate the groups (see Table 1). ESAT-6 reactivity, both using ICS and IFN-γ measurement in the supernatant was not found in all TB patients although the diagnosis of TB was fully confirmed by clinical presentation, X-ray, smear, and/or biopsy. Also, unexpectedly, ESAT-6 reactivity occurred in a few of the control patients, suggesting they might have been latently infected. As with tuberculin induced responses, the mere presence/absence or size of T-cell responses to ESAT-6 alone would not have been helpful for the diagnosis of active pulmonary TB in this cohort (see Table 1). Table 3 shows responder frequencies in the different TB related tests used in this study, for the purpose of this comparison only, IFN-γ levels exceeding 0.1 IU/ml were arbitrarily considered a ‘positive’ result.

**Table 3:** Responder frequencies^a^ in different TB related tests in the blind study.

Patient group	TST≥5 mm	tuberculin in vitro^b^ ICS vs. supernatant	ESAT-6 in vitro^c^ ICS vs. supernatant
smear and/or culture positive TB (n = 12)	5/7	12/12 vs. 12/12	5/11 vs. 5/11
smear and culture negative TB (n = 10)	6/9	7/10 vs. 5/10	1/10 vs. 1/10
≥1 year after beginning of TB treatment (n = 8)	5/7	9/9 vs. 8/8	4/9 vs. 4/8
Control patients (n = 17)	0/12	10/17 vs. 9/17	3/16 vs. 3/15

a not all test were performed in all individuals, numbers refer to positive test results per tested individuals

b frequency of tuberculin-reactive CD4 T-cells (IFN-gamma) above 1/10,000 or IFN-gamma above 0.1 IU/ml (arbitrary value)

c frequency of ESAT-6 reactive CD4 T-cells (IFN-gamma) above 1/10,000 or IFN-gamma above 0.1 IU/ml (arbitrary value)

### Changes in treated patients occur very slowly

**Figure 5:**
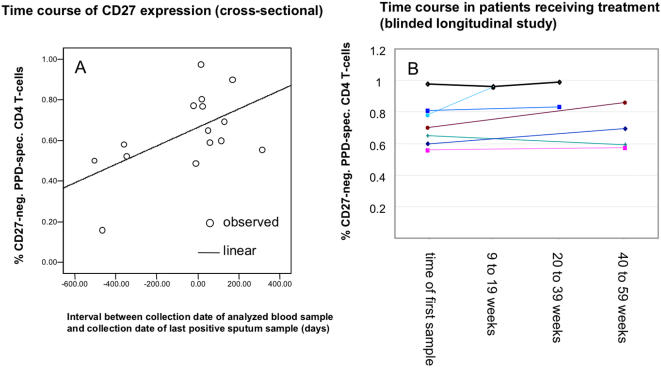
CD27 expression on tuberculin-reactive CD4 T-cells reverts very slowly . A: CD27-expression on tuberculin-reactive CD4 T-cells was plotted against the length of the interval between the collection date of the analyzed blood sample and the collection date of the last positive sputum sample (all data from blinded study). Negative values indicate that sputum was already smear and culture negative when tuberculin-reactive CD4 T-cells were analyzed, positive values indicate that sputum samples were still positive and remained so for the indicated time. B: serial measurements in 7 individuals (data analysis blinded).

In group III, 3 of 4 patients with-initially-smear positive TB had percentages of CD27-neg. tuberculin-responsive CD4 T-cells above 50%, still one year after therapy, indicating that CD27 expression was not restored quickly after treatment (see Fig. 4A). However, the interval between the collection date of the analyzed blood sample and that of the last positive sputum sample (culture and/or microscopy) in each patient correlated albeit weakly with the percentage of CD27-negative tuberculin-reactive CD4 T-cells (0.562, p = 0.029) (Figure 5 A). The collection time of the last positive sputum sample is the closest surrogate for the last time point when TB was not contained with subsequent cultures turning out negative. If free access of mycobacteria to the bronchial mucosa was involved in driving the continued differentiation of tuberculin specific CD4 T-cells, this time point would be considered a turning point from which onwards differentiation may stop or revert. To further explore this hypothesis using longitudinal rather than cross-sectional data, additional follow-up samples from patients with smear and/or culture positive pulmonary TB were requested, and assessed, as before, in a blinded manner. Seven samples from time points between nine weeks and one year after the first sample were obtained. However, we found that CD27-expression on the tuberculin-reactive CD4 T-cells was fairly stable over the measured treatment intervals up to one year (Figure 5 B) which did not confirm the idea of reversion of this parameter, at least not within the time frame of one year.

## Discussion

Our results have demonstrated that CD27-expression on ex-vivo short term stimulated tuberculin specific CD4 T-cells is a highly discriminating biomarker for active pulmonary TB. It will also have utility for tracing TB contacts with latent TB infection. In this cohort of patients and controls the phenotype of tuberculin-reactive T-cells was more informative than quantitative measures of reactivity or target protein specificity.

While paradigms for compartmentalizing antigen dependent T-cell differentiation based on surface markers and functionality have been an area of major interest for a number of years [14], [20], [21], patterns of T-cell differentiation have not previously been used to help making a clinical diagnosis. In TB, such an approach appears very attractive, in particular in a BCG vaccinated population, where an immune response to tuberculin is not diagnostic of infection. Our approach utilizes the fact that BCG vaccination is a single stimulus and will drive T-cell differentiation only to a limited extent. Only once additional exposure to TB occurs will additional differentiation occur. It is exactly that increment of differentiation which leads us to the diagnosis, exploiting the fact that boosting of BCG-induced responses *exclusively* occurs by subsequent exposure to TB.

The degree of T-cell differentiation will likely depend on the circumstances, length, and frequency of exposure, and also the efficiency of the T-cell response in containing infection. These are obviously interdependent factors. In the 6 BCG vaccinated unexposed controls shown in Fig. 3, the percentage of CD27-negative tuberculin-reactive CD4 T-cells did not exceed 35%. This may be the degree of differentiation expected in response to BCG vaccination alone. Fig. 3 suggests that there is more differentiation in individuals with continued exposure. Highly exposed individuals show levels of CD27-neg. tuberculin-reactive CD4 T-cells similar to those found in latent TB infection and even acute smear and/or culture positive TB. It is not known whether contact with TB is required in order to maintain a minimum of tuberculin-reactive CD4 T-cells throughout life, however, our data suggests there are gradual differences in exposure related differentiation, rather than everything or nothing. It may well be that a minimum of exposure helps to maintain a minimum response, whereas a higher degree or frequency of exposure (such as in hospital personnel) will push differentiation.

The fact that we did not observe more CD27-neg. tuberculin-reactive CD4 T-cells in patients with smear and culture negative TB than in control patients is of interest, since there was a clear distinction between unexposed donors and individuals with latent TB (Fig. 3). Smear negative TB would be expected to have developed from TB that was once latent or active, so that reversion of the predominantly CD27-neg. phenotype must have occurred with time. Before active smear and culture negative TB was diagnosed (usually initiated by doing a chest X-ray for other reasons) the infection may have been contained and inactive for years. All patients in this group had typical findings on chest films, and 8 out of 10 had positive biopsy results. In two patients the diagnosis was partly based on a therapy response (reduction of lesions), in the remaining patients disease activity was not demonstrated.

Recently it was shown in a TB mouse model that CD27-neg. lymphocytes isolated from lung produced more IFN-γ following stimulation with tuberculin than their CD27-pos. counterparts, which agrees with our findings in humans [19]. In smear and/or culture positive TB more than half of the tuberculin-reactive IFN-γ producing CD4 T-cells had lost CD27. Direct exposure of bronchus associated lymphatic tissue (BALT) to free *mycobacteria* may play an important role in triggering and sustaining T-cell differentiation [22]. This might be mediated by alveolar macrophages that are activated via Toll Like Receptors or related signalling pathways [23]–[25]. This would be considered an “uncontrolled” traffic of pathogens, causing responses that are different in quality from those occurring as pathogens are transported in and out of organized lesions which are under the control of the immune system. We tentatively conclude that the percentage of CD27-expressing tuberculin-reactive CD4 T-cells is most likely a reflection of the degree of exposure of the bronchus associated lymphatic system to free *mycobacteria*l antigens.

The combination of low CCR7 but high CD28 expression that we observed on all tuberculin-reactive CD4 T-cells was recently associated with lung homing, which is indeed a prominent feature of tuberculin responsive CD4 T-cells [26].

The finding that ESAT-6 reactivity was not a good marker for the diagnosis of active TB is in agreement with previous reports on acute TB patients and controls for example in Brazil and The Gambia [7], [27], indicating that ESAT-6 responses are higher in latent TB but not active TB than in controls. Of note, ESAT-6 reactivity is normally measured in epidemiological studies to detect latent TB infection and not used to diagnose acute TB [1], [4].

In order to rule out any bias related to subjective interpretation of flow-cytometry data the second part of the study was performed in a blinded fashion. This has fully confirmed our initial findings and demonstrated that gating of CD27-negative T-cells is a robust procedure. The fact that in some patients less than 50 IFN-γ producing cells were available for assessing the percentages of CD27-pos. and CD27-neg. cells reflects that our samples were stretched somewhat to accommodate several stimuli and antibody panels per sample in this pilot study. If only tuberculin had been used as a stimulus in combination with just one antibody panel, we would have had largely sufficient numbers of events in all TB patients.

In conclusion, our results argue that the phenotype of TB specific T-cells measured at the single cell level has considerable diagnostic potential and is more relevant than response quantity. A similar change of paradigm has already taken place in the HIV field [28], [29].

Since results can be obtained in less than 24 hours, this method could have utility in situations where diagnostic decisions are a matter of urgency. Smear and/or culture positive TB is a hazard to society and unnecessary hospitalizations are costly. It is, therefore, important to identify these patients rapidly. In this respect, our new assay is a considerable advance and calls for more research into using T-cell differentiation in diagnostic procedures in TB and other fields.
